# Targeted next-generation sequencing using bronchoalveolar lavage fluid samples for diagnosing pulmonary infections: a single-center retrospective study

**DOI:** 10.3389/fmicb.2025.1671819

**Published:** 2025-10-13

**Authors:** Jinbao Huang, Ling Ye, Heng Weng, Meiqin Jiang, Ying Lin, Hongyan Li, Baosong Xie

**Affiliations:** ^1^Shengli Clinical Medical College of Fujian Medical University, Fuzhou, China; ^2^Department of Pulmonary and Critical Care Medicine, The Affiliated People’s Hospital of Fujian University of Traditional Chinese Medicine, Fuzhou, China; ^3^Department of Clinical Laboratory Medicine, The Affiliated People’s Hospital of Fujian University of Traditional Chinese Medicine, Fuzhou, China; ^4^Department of Critical Care Medicine, The Affiliated People’s Hospital of Fujian University of Traditional Chinese Medicine, Fuzhou, China; ^5^Department of Pulmonary and Critical Care Medicine, Fujian Provincial Hospital, Fuzhou, China

**Keywords:** targeted next-generation sequencing, bronchoalveolar lavage fluid, pathogen, diagnosing, pulmonary infection

## Abstract

**Objectives:**

To evaluate the diagnostic value of targeted next-generation sequencing (tNGS) of bronchoalveolar lavage fluid (BALF) samples from patients with pulmonary infections (PIs).

**Methods:**

This retrospective study included 110 patients diagnosed with suspected PIs, who underwent tNGS of BALF samples between February 2023 and January 2025. Conventional microbiological tests (CMTs), traditional culture, and tNGS were simultaneously performed, and the diagnostic efficiencies of the three PI methods were compared.

**Results:**

A total of 110 BALF samples were obtained from 110 patients, including 80 with PIs and 30 without PIs. The detection sensitivities of tNGS, culture, and CMTs for the diagnosis of PIs significantly differed (*P* < 0.001). Further analysis showed that the sensitivity of tNGS was higher than those of culture (*P* < 0.001) and CMTs (*P* = 0.003). The specificity of the culture was higher than those of tNGS (*P* < 0.001) and CMTs (*P* < 0.001). However, the accuracy of culture was lower than those of tNGS (*P* < 0.001) and CMTs (*P* = 0.022), and the accuracy of CMTs was lower than that of tNGS (*P* = 0.022). Additionally, the area under the receiver operating characteristic curve for tNGS was better than that for CMTs (0.627 vs. 0.510). Among all causative pathogens, the bacteria were the most prevalent ones, of which the most commonly detected pathogens were *Streptococcus pneumoniae* (15.2%, 10/66), *Pseudomonas aeruginosa* (13.6%, 9/66), atypical pathogens (including *Chlamydia psittaci*, *Chlamydia pneumoniae*, and *Mycoplasma pneumoniae*) (13.6%, 9/66), *Haemophilus influenzae* (10.6%, 7/66), *Klebsiella pneumoniae* (10.6%, 7/66), and *Mycobacterium tuberculosis* complex (10.6%, 7/66). The most commonly detected fungi were *Pneumocystis jirovecii* (40.0%, 4/10) and *Aspergillus* (40.0%, 4/10), and the most commonly detected viruses were influenza virus A/B (55.6%, 10/18), and severe acute respiratory syndrome coronavirus-2 (27.8%, 5/18). The BALF tNGS results led to changes in the clinical plans of 50 (45.5 %) patients. However, the existing clinical management protocol was maintained in 28 patients (25.5 %) because the tNGS results supported the current diagnosis and management. Additionally, 32 (29.1 %) patients underwent adjustment of the clinical regimen or an unchanged clinical regimen based on empirical judgment and/or CMT results.

**Limitations:**

This study had certain limitations, such as its retrospective design, relatively low specificity, and difficulty in identifying colonizing microorganisms. Through forward-looking in-depth research, the continuous accumulation of clinical experience, or the integration of artificial intelligence, tNGS will enable more precise and efficient management strategies for PIs.

**Conclusion:**

The sensitivity and accuracy of tNGS were better than those of culture and CMTs. tNGS results were critically associated with the development of clinical treatment plans for most patients. tNGS can be used as a rapid and accurate auxiliary diagnostic method, along with CMTs, for PIs.

## Introduction

Lower respiratory tract infections have led to high morbidity and mortality worldwide (GBD 2021 Lower Respiratory Infections and Antimicrobial Resistance Collaborators, 2024). Owing to the wide variety of pathogens, the quick and accurate detection of the target pathogen and precise anti-infection treatment have always been major challenges in clinical practice. Although conventional microbiological tests (CMTs) performed in laboratories encompass various methods, these techniques can only identify approximately 40 % of pathogens and are associated with a series of limitations (Qu et al., 2022; Shi et al., 2022). Traditional staining and culture techniques are still widely used for the detection of common pathogens; however, these methods have limitations, such as low sensitivity, prolonged culture periods, and the requirement for stringent or difficult culture conditions for certain pathogens (Xu et al., 2024; Gu et al., 2025). Molecular methods such as polymerase chain reaction (PCR) and its derivative technologies, gene chip technology, and immunoassays have become some of the leading methods for the diagnosis of pulmonary infections (PIs). However, these methods require the assumption of diagnosis and are typically limited to the detection of specific pathogen categories, exhibit a narrow detection spectrum, and are susceptible to potential false-negative results (Azad et al., 2022; Cai et al., 2024; Xu et al., 2024; Gu et al., 2025). Consequently, traditional methods for diagnosing pathogens do not adequately address the requirements of clinical practice (Qu et al., 2022; Shi et al., 2022; Liu et al., 2024).

Metagenomic next-generation sequencing (mNGS) has recently emerged as a novel technology for pathogen diagnosis (Han et al., 2019). This method is characterized by its short detection time and broad detection spectrum (Gökdemir et al., 2022), enabling the precise identification of various pathogens, including bacteria, fungi, viruses, and parasites (Indelli et al., 2021). These capabilities represent substantial improvements over traditional detection methods. Although this technology can simultaneously detect approximately 20,000 different types of microorganisms (Huang J. et al., 2023), its complexity and high cost restrict its routine clinical use (Wang and Liu, 2020; Liu et al., 2024), hindering its widespread adoption and application.

Targeted next-generation sequencing (tNGS) was recently developed to identify the most prevalent pathogen types in clinical settings. This advanced technique leverages a panel of predesigned multiplex pathogen primers (multiplex PCR) in conjunction with high-throughput sequencing techniques to identify pathogens. Highly multiplex PCR, as a component of the tNGS workflow, represents a cost-effective and rapid molecular detection approach (Lou et al., 2025); nevertheless, its sensitivity is lower than that of mNGS with higher throughput (Lou et al., 2025). By integrating the strengths of these two approaches, tNGS enables more accurate and efficient pathogen detection. In addition to exhibiting enhanced sensitivity, this approach markedly reduces turnaround times (as low as 14.5–16 h) (Cai et al., 2024; Ye et al., 2024) and detection costs (one-quarter to one-half that of mNGS) (Chen et al., 2024; Ye et al., 2024), thereby mitigating the economic burden on patients (Li et al., 2021; Huang C. et al., 2023). A study on bloodstream infections (BSIs) demonstrated that the sensitivity of blood tNGS for diagnosing BSIs was significantly higher than that of blood cultures (91.3 % vs. 23.2 %, *P* < 0.001) and mNGS (91.3 % vs. 69.6 %, *P* = 0.001) (Cai et al., 2024). Li et al. (2023) reported that the diagnostic sensitivity of tNGS for central nervous system infections was markedly higher than that for conventional cerebrospinal fluid cultures and smears (81.8 % vs. 13.6 %). Another study involving adult patients with hematological malignancies and suspected infections demonstrated that pathogen detection using multiple specimen types through tNGS exhibited significantly higher sensitivity (69.7 % vs. 35.9 %), negative predictive value (NPV) (48.2 % vs. 42.4 %), and accuracy (66.5 % vs. 56.5 %) than CMTs (Xu et al., 2024). In a study evaluating 130 cases of severe pneumonia, the concordance rate between tNGS results and clinical diagnosis exceeded 70 %, and the detection of pathogenic microorganisms using tNGS was consistent with culture, mNGS, and real-time quantitative PCR findings (Zhang et al., 2024). Moreover, in specific respiratory samples, tNGS exhibited a high detection sensitivity for pathogens and maintained a high sensitivity (70.8%–95.0%) (Chen et al., 2024).

Currently, reports describing its diagnostic value for multiple pathogens in PIs are limited, particularly concerning the absence or lack of representative data on diagnostic specificity (Zhang et al., 2023; Liu et al., 2024; Guo et al., 2025). In addition, it is difficult to determine the pathogenic potential of the detected microorganisms. Therefore, in this study, we retrospectively analyzed tNGS results from bronchoalveolar lavage fluid (BALF) samples of 110 patients with suspected PIs to assess their diagnostic accuracy and clinical value, compared with conventional methods.

## Materials and methods

### Patients

Data of patients who underwent tNGS between February 2023 and January 2025 at the Affiliated People’s Hospital of Fujian University of Traditional Chinese Medicine were retrospectively analyzed. The inclusion criteria were as follows: (1) age ≥ 18 years; (2) patients with a preliminary diagnosis of PIs; and (3) patients with BALF samples that had undergone tNGS DNA and RNA detection and CMTs. The exclusion criteria were as follows: (1) unclear diagnosis; (2) specimens other than BALF; (3) unqualified quality control of the tNGS detection specimens; and (4) incomplete medical data.

The diagnostic criteria for PIs and non-PIs were established through a comprehensive clinical analysis, incorporating symptoms, physical examinations, laboratory tests, imaging abnormalities, and evidence of effective targeted drug treatments. The details of the analyses are described in previous studies (Huang J. et al., 2023; Liu et al., 2024). This study was conducted in accordance with the Declaration of Helsinki and approved by the Ethics Committee of the Affiliated People’s Hospital of Fujian University of Chinese Medicine. Considering the retrospective nature of the study, the requirement for informed consent was waived, and all the data were anonymously analyzed.

### Conventional microbiological detection

The CMTs included fungal and bacterial cultures and smears, Mycobacterium tuberculosis (MTB) complex DNA, GeneXpert MTB/rifampicin (RIF), *Acetobacter* smears, PCR analysis of respiratory pathogens [such as *Mycoplasma*, severe acute respiratory syndrome coronavirus-2 (SARS-CoV-2), influenza A, influenza B, adenovirus, and respiratory syncytial virus], influenza A and influenza B antigens, cryptococcal antigen (CrAg), 1, 3-β-D-glucan (G) and galactomannan (GM) antigens, humoral cell Hexamidosilver staining for *Pneumocystis jirovecii* (PJ), and serum pathogen IgM and IgG antibodies. The test samples included blood, sputum, and BALF.

### Construction of tNGS panel and specimen collection

The internal tNGS panel was designed to cover 363 microbial targets, including 208 bacteria, 54 fungi, 64 viruses, 18 parasites, and 19 special pathogens such as *Rickettsia*, *Chlamydia*, *Mycoplasma*, *Spirochaeta*, and *Ureaplasma*. These microorganisms include the most common respiratory pathogens and the less common but highly virulent pathogens (Lei et al., 2025).

BALF specimens (≥ 5 mL) were collected from patients who underwent bronchoscopy. The specimens were immediately transported to the laboratory in cold storage for testing. If the specimen could not be sent for timely testing, it was stored at 2 °C –8 °C for a maximum duration of 24 h. All specimens were subjected to NGS detection and pathogen analysis at an independent third-party testing facility (Hangzhou Matridx Biotechnology Co., Ltd, Hangzhou, China).

### tNGS testing procedure

Nucleic acids, including DNA and RNA, were extracted from 1.0 mL BALF samples using a total nucleic acid extraction kit (Cat.MD049T; MatriDx Biotech Corp. Hangzhou, China). After DNA/RNA extraction, multiplex PCR was performed to selectively amplify and enrich specific pathogen target sequences using a pathogen-targeting multiplex amplification kit (Cat.MD061X; MatriDx Biotech Corp. Hangzhou, China). Total DNA/RNA library preparation kit (Cat.MD001T; MatriDx Biotech Corp. Hangzhou, China) was used for library preparation, which included enzymatic fragmentation of genomic DNA/RNA, end repair, terminal adenylation, and adaptor ligation, followed by purification. Library concentrations were quantified by real-time PCR using the KAPA system, and sequenced using an Illumina NextSeq 550 sequencer (San Diego, California, United States). A total of one million reads (minimum 0.05 M) were obtained for each sample for bioinformatic analysis. All commercial kits were subjected to rigorous internal validation in a laboratory before their application in clinical practice. The test results demonstrated stability and reliability, thereby satisfying the validation criteria. In addition, three repeatability tests were performed, and the results exhibited consistent performance, fulfilling the validation requirements.

### Quality control and bioinformatic pipeline

Negative and positive controls were concurrently used to monitor and assess potential contamination, as described in our previous study (Huang J. et al., 2023), to ensure the quality of each sequencing batch. The entire experimental process involved only a single PCR step and utilized fragmented library construction, thereby substantially reducing the risk of contamination during subsequent purification procedures. Furthermore, several laboratory-specific background microbial databases have been developed to track microbial contamination in reagents and experimental environments, thereby effectively preventing and mitigating microbial contamination.

The primary steps of bioinformatics analysis were conducted as follows: (1) the short (length < 35 bp), low-quality, and low complexity reads, as well as the adapter sequences were excluded from raw sequenced reads; (2) the host sequences were eliminated by aligning them to the human-specific database in NCBI (GRCh38.p13), utilizing Bowtie2 (version 2.3.5.1); and (3) the clean reads were then aligned to an in-house microbial database, including the NCBI nt database, GenBank, and mNGS data, for taxonomic classification at the species level.

### tNGS reporting criteria

Similar to the mNGS detection method previously described (Huang J. et al., 2023), the microbial reads identified from the library were reported according to strict reporting criteria. The key points included ensuring that sequencing data met quality control standards and that specimens failing to meet negative control criteria were removed to maintain a clear differentiation between true pathogenic bacteria and background environmental contaminants.

In addition to presenting the sequencing results, the relative abundance was estimated in the pathogen report. A notable increase in the relative abundance generally indicates considerable growth in the population of microbial species within the sample. Based on this, the test report generally adopts a relative abundance of ≥ 50 % as the threshold for common colonizing microorganisms, such as *Streptococcus constellatus* and *Finegoldia magna* in the respiratory tract, to indicate potential pathogenic significance.

### Interpretation of pathogenicity of the detected microorganisms in the tNGS report

The results of the tNGS report were independently interpreted by three professionals of infectious diseases or microbiological test analysis and with knowledge of bioinformatics. The composite reference standard for determining the actual pathogen included results from all laboratory tests (including tNGS), radiological characteristics, pathological findings, host factors, clinical symptoms, therapeutic responses, and comprehensive clinical evaluation. For discrepancies in pathogen identification, a panel of three experts reached a consensus following a comprehensive group discussion and analysis (Huang J. et al., 2023).

Notably, when interpreting the tNGS report, if the pathogenic significance was comparable, a higher number of reads for a particular pathogen suggested a greater likelihood of it being the causative agent of the infection. Additionally, changes in relative abundance can serve as valuable references for clinical decision-making, particularly for colonizing anaerobic microorganisms. Similar to the criteria in previous studies (Blauwkamp et al., 2019; Guo et al., 2021), the interpretation results of pathogenicity were classified into four categories (definite, probable, possible, and unlikely): (1) definite: the pathogens identified through tNGS were entirely consistent with the findings detected by CMTs within 7 days of specimen collection, as confirmed by pathological examination and/or comprehensive clinical evaluation; (2) probable: based on a comprehensive assessment incorporating clinical findings, imaging results, and laboratory tests, etc., the pathogen detected by tNGS was highly likely to be the causative agent of PIs; (3) possible: the pathogen identified by tNGS exhibited potential pathogenicity and aligns with the clinical features, but an alternative explanation was more plausible; (4) unlikely: the pathogen detected by tNGS possessed potential pathogenic properties but was inconsistent with the findings of the comprehensive clinical assessment. Definite and probable results were judged as causative agents for the final diagnosis, whereas possible and unlikely results were judged as non-causative agents for the final diagnosis.

### Statistical analysis

Statistical software (SPSS 19.0; IBM SPSS Inc., Chicago, IL, United States) was used for the data analysis. Missing data were examined and found to be completely random and minimal, comprising < 2 % of all data points. Therefore, listwise deletion was employed. Measurement data are expressed as median (interquartile range). Count data are expressed as percentages (%), and the diagnostic performance of the different testing methods (tNGS, traditional culture, and CMTs) is expressed in terms of sensitivity, specificity, accuracy, NPV, and positive predictive value. The diagnostic efficacy was compared using the chi-squared test. Prior to the analysis, the assumptions of the chi-squared test were verified. As more than 20 % of the cells had an expected count below 5, Fisher’s exact test was used instead of the standard chi-square test. The area under the curve (AUC), which was used to evaluate and compare the performances of tNGS and CMTs, was determined using receiver operator characteristic (ROC) curve analysis. Statistical significance was set at *P* < 0.05.

## Results

### Characteristics of the enrolled patients

**FIGURE 1:**
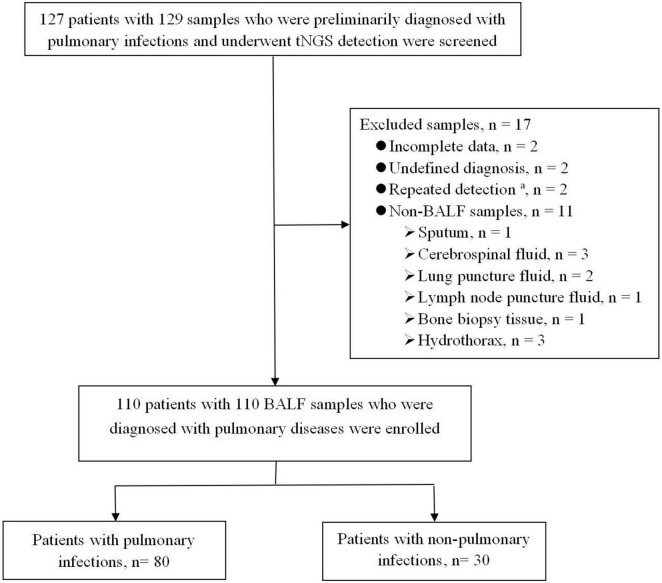
Flowchart of enrolled patients. BALF, bronchoalveolar lavage fluid; tNGS, targeted next-generation sequencing. ^a^The same patient was tested multiple times, and the results of the first BALF specimen were selected.

Between December 2023 and January 2025, 129 human specimens from 127 patients were tested by tNGS. Finally, 110 BALF samples from 110 patients initially diagnosed with PIs met the inclusion criteria (Figure 1). The enrolled patients included 93 patients from the respiratory department, eight from the comprehensive intensive care unit, eight from the hematology department, and one from the cardiology department.

As shown in Table 1, 110 patients included 80 with PIs and 30 without PIs, and 86 patients (78.2 %) had underlying diseases, including 50 with immunosuppressive diseases. The median age was 59 (49, 69) years and half of the patients were female.

**TABLE 1:** Comparison of clinical characteristics between patients with pulmonary infections and those with non-pulmonary infections.

Clinical characteristics	Total (*n* = 110)	Pulmonary infections (*n* = 80)	Non-pulmonary infections (*n* = 30)	*P*
Age, mean (range), years	59 (49, 69)	60 (49, 71)	58 (49, 63)	0.785
Sex, female, *n* (%)	55 (50.0)	40 (50.0)	15 (50.0)	1.000
Underlying disease, *n* (%)	86 (78.2)	63 (78.8)	23 (76.7)	0.794
Immunosuppressive disease, *n* (%)	50 (45.5)	36 (45.0)	14 (46.7)	0.544
Community-acquired infection	—	66 (82.5)	—	—
Hospital-acquired infection	—	13 (16.2)	—	—
Ventilator-associated infection	—	1 (1.3)	—	—
Antibiotic use before tNGS, *n* (%)	86 (78.2)	69 (86.3)	17 (56.7)	0.002
White blood cell count, 10^9^/L	6.85 (5.10, 10.40)	7.10 (5.20, 10.80)	6.55 (4.88, 9.38)	0.477
Neutrophil count, 10^9^/L	4.85 (3.20, 7.30)	5.15 (3.20, 7.68)	4.25 (3.20, 6.90)	0.452
Leukomonocyte count, 10^9^/L	1.10 (0.68, 1.70)	1.10 (0.60, 1.70)	1.35 (1.00, 1.75)	0.124
C-reactive protein, mg/L	24.48 (5.59, 75.76)	38.21 (8.69, 109.00)	10.05 (1.79, 41.13)	0.002
Procalcitonin, ng/mL	0.07 (0.04, 0.27)	0.09 (0.05, 0.43)	0.05 (0.03, 0.11)	0.002

—, data not available. Data are presented as *n* (%) or mean (range). Significance was determined using the chi-square test or non-parametric rank sum test.

### Results of tNGS testing

Of the 110 BALF samples that underwent tNGS, 71 (64.5 %) were identified as disease-causing pathogens. In patients with PIs, the sensitivity of pathogen detection did not significantly differ between those who received antibiotics before tNGS and those who did not [88.4 % (61/69) vs. 90.9 % (10/11), *P* > 0.05].

**FIGURE 2:**
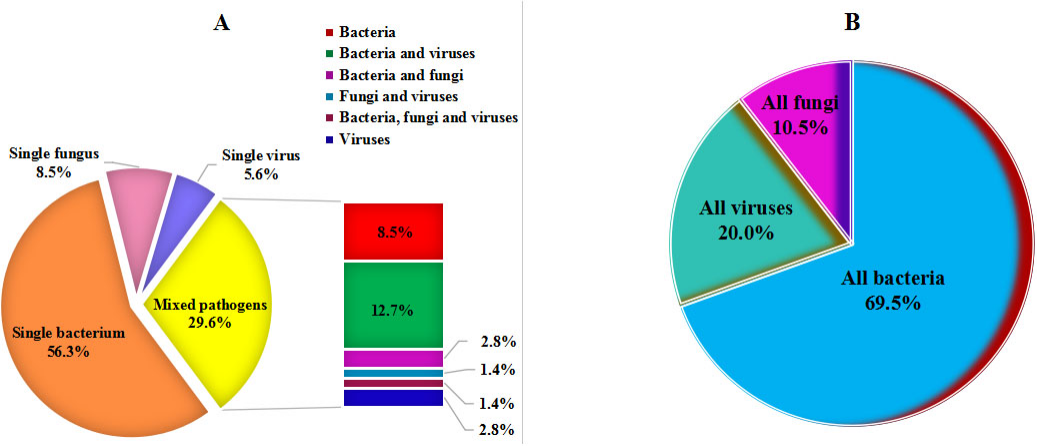
Disease-causing pathogens detected by targeted next-generation sequencing. **(A)** Categorization of single pathogen and mixed pathogens. **(B)** Classification of all pathogens.

**FIGURE 3:**
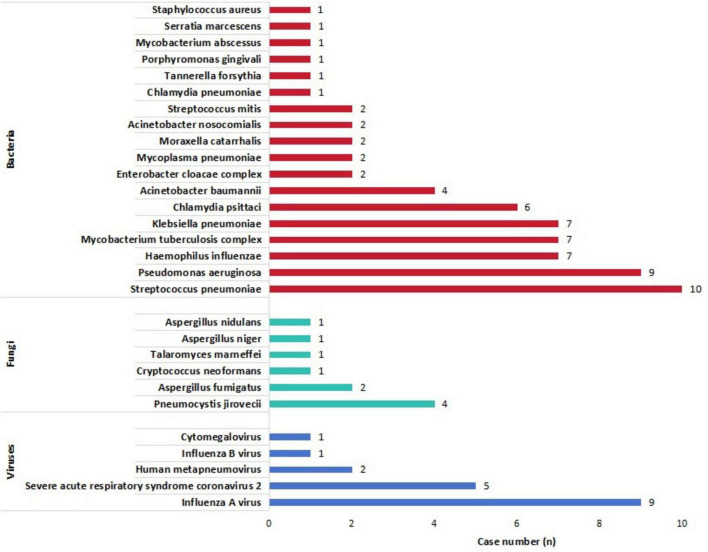
Detailed distribution of all disease-causing pathogens detected by targeted next-generation sequencing.

Among the 71 patients with PIs, the results of pathogens identified by tNGS are illustrated in Supplementary Tables 1, 2 and Figures 2, 3. In addition, 119 non-causative microorganisms were simultaneously detected in 71 patients with PIs (52 cases) and non-PIs (19 cases) (Supplementary Figures 1, 2). The most commonly detected pathogens were various viruses (50 %, 58/116), such as human herpesvirus (75.9 %, 44/58) and human parainfluenza virus (13.8 %, 8/58). Among all the non-pathogenic viruses, 11 (19.0 %) were detected in eight (26.7 %) patients with non-PIs.

### Comparison of different testing methods for PIs tNGS

Significant differences were observed in the sensitivities of tNGS, culture, and CMTs for PI diagnosis (*P* < 0.001). In the pairwise comparisons analysis, the sensitivity of tNGS was higher than those of culture (*P* < 0.001) and CMTs (*P* = 0.003); the sensitivity of CMTs was also higher than that of culture (*P* < 0.001). The sensitivity of tNGS combined with CMTs for detecting PIs was 95.0 % (76/80). The detection specificity and accuracy of tNGS, culture, and CMTs for diagnosis of PIs significantly differed (*P* < 0.001 and *P* < 0.001, respectively). Further analysis showed that the specificity of the culture was higher than those of tNGS (*P* < 0.001) and CMTs (*P* < 0.001); however, no significant difference was observed in the specificity between tNGS and CMTs (*P* > 0.05). On the contrary, the accuracy of the culture was lower than those of tNGS (*P* < 0.001) and CMTs (*P* = 0.022), and the accuracy of CMTs was lower than that of tNGS (*P* = 0.022) (Table 2).

**TABLE 2:** Comparison of different diagnostic methods for pathogen identification.

Diagnostic performance	tNGS (*n* = 110)	Culture (*n* = 110)	CMTs (*n* = 110)	*P*
Sensitivity %	88.8 (79.2, 94.4)*	22.5 (14.2, 33.5)	68.8 (58.5, 76.6)	< 0.001
Specificity %	36.7 (20.5, 56.1)	96.7 (80.9, 99.8)	33.3 (17.9, 52.9)	< 0.001
Accuracy %	74.5 (66.4, 82.7)	42.7 (33.5, 52.0)	59.1 (49.0, 68.3)	< 0.001
PPV %	78.9 (68.8, 86.5)	94.7 (71.9, 99.7)	73.3 (61.7, 82.6)	0.134
NPV %	55.0 (32.0, 76.2)	31.9 (22.7, 42.6)	28.6 (15.2, 46.5)	0.103

*95% confidence interval. Data are presented as *n* (%). Significance was determined using the Fisher’s exact test. CMTs, conventional microbiological tests; NPV, negative predictive value; PPV, positive predictive value; tNGS, targeted next-generation sequencing.

Targeted next-generation sequencing showed a higher detection sensitivity than CMTs for both single- and mixed-pathogen infections (90.2 %, 46/51 vs. 64.7%, 33/51, *P* = 0.004; 84.0%, 21/25 vs. 32.0%, 8/25, *P* < 0.001, respectively). tNGS also showed superior detection capability, compared with CMTs, for infections caused by bacteria, atypical pathogens, and viruses (*P* < 0.05). However, the sensitivity of fungi was not significantly different between tNGS and CMTs (*P* > 0.05), and the sensitivity of MTB was 100 % for both methods (Table 3).

**TABLE 3:** Comparison of pathogen-identification ability between tNGS and CMTs.

The number of pathogens*	tNGS (*n*, %)	CMTs (*n*, %)	*P*
Bacterium (*n* = 72)	66 (91.7)	47 (65.3)	< 0.001
Atypical pathogens ^a^ (*n* = 9)	9 (100)	1 (11.1)	< 0.001
MTB (*n* = 7)	7 (100)	7 (100)	—
Fungus (*n* = 13)	10 (76.9)	8 (61.5)	0.673
Virus^b^ (*n* = 18)	17 (94.4)	11 (61.1)	0.019

*The number of pathogens refers to the total number of specific pathogens in single and mixed infections.

^a^Atypical pathogens include *Chlamydia psittaci*, *Chlamydia pneumoniae*, and *Mycoplasma pneumoniae.*

^b^Viruses include RNA viruses (influenza A virus, influenza B virus, severe acute respiratory syndrome coronavirus 2, and human metapneumovirus), and a DNA virus (cytomegalovirus). Data are presented as *n* (%). Significance was determined using the chi-squared test or Fisher’s exact test. tNGS, targeted next-generation sequencing; CMTs, comprehensive microbial tests; MTB, *Mycobacterium tuberculosis* complex; NTM, non-tuberculous mycobacteria; —, not available.

### ROC curves of tNGS and CMTs for diagnosis of PIs

**FIGURE 4:**
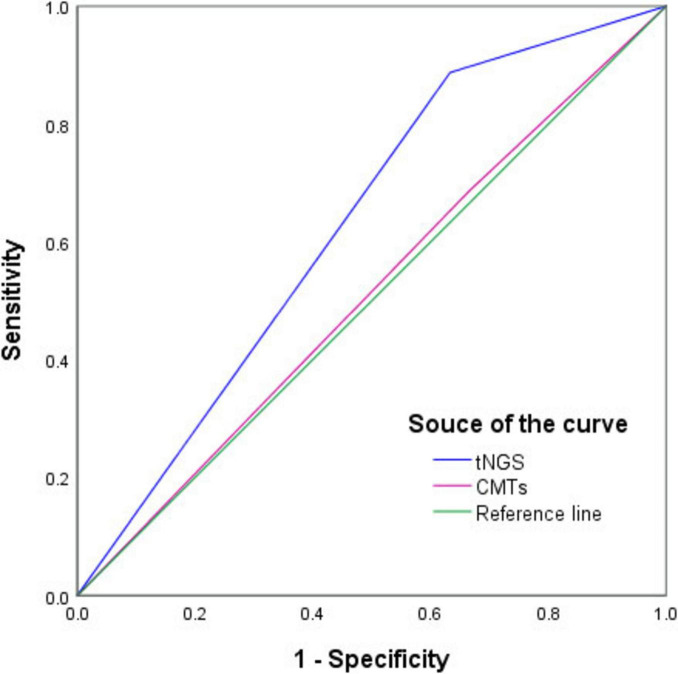
Receiver operating characteristic curve of targeted next-generation sequencing (tNGS) and conventional microbiological tests (CMTs) for diagnosis of pulmonary infections. In comparison with the composite reference standard for clinical diagnosis, the sensitivity and specificity of tNGS and CMTs were 88.8% (95% CI, 79.2%–94.4%)/36.7% (95% CI, 20.5%–56.1%) and 68.8% (95% CI, 58.5%–76.6%)/33.3% (95% CI, 17.9%–52.9%), respectively.

As shown in Figure 4, the diagnostic values of tNGS and CMTs for PIs were compared using ROC curves, and the AUCs were 0.627 [95 % confidence interval (CI), 0.752–0.502; *P* = 0.041] and 0.510 (95 % CI, 0.632–0.388; *P* = 0.867), respectively.

### Adjustment of therapeutic strategies on the basis of tNGS results and clinical outcomes

As shown in Table 4, the BALF tNGS test findings were associated with changes in the clinical plans of 50 patients (45.5 %). In contrast, the existing clinical management protocol was maintained in 28 patients (25.5%) regardless of the tNGS results because the tNGS results supported the current diagnosis and management. Additionally, 32 patients (29.1 %) received an adjusted or unchanged clinical regimen based on empirical judgment or CMTs.

**TABLE 4:** Adjustment of clinical management and outcomes.

Adjustment type	No. (%)
Adjustment was indicated on the basis of the results of tNGS testing	50 (45.5)
Identified infectious agents and initiated anti-infective therapy	18 (34.6)
Identified infectious agents and escalated anti-infective therapy	21 (42.0)
Identified infectious agents and de-escalated anti-infective therapy*	1 (2.0)
Ruled out infection	10 (20.0)
Discontinued antibiotics and initiated non-infectious therapy	6 (60.0)
Discontinued antibiotics and conducted follow-up observation	2 (20.0)
Initiated non-infectious therapy	2 (20.0)
No adjustment was indicated on the basis of the results of tNGS testing	28 (25.5)
Supported the original treatment regime	25 (89.3)
Supported non-infection and conducted follow-up observation	1 (3.6)
Identified infectious agents but targeted treatment could not be initiated due to lack of effective drugs	2 (7.1)
Therapeutic regimen changes or maintenance were not based on tNGS testing	32 (29.1)

*De-escalation did not include complete discontinuation of antibiotics.

Among the 110 patients, most were cured or improved, with just nine deaths recorded (8.2 %). Among these nine patients, five (55.6 %) underwent an adjustment of their anti-infection regimens based on the tNGS results (Supplementary Table 3). All five patients had severe pneumonia with multiple complications and received upgraded antibiotics. Of the five patients who died, four were infected with multidrug-resistant *Acinetobacter baumannii*, and two died from reinfection, although the infection was initially controlled. One patient was infected with multidrug-resistant *Klebsiella pneumoniae* and died after the infection worsened owing to repeated aspiration caused by refusal to undergo tracheotomy after infection control.

## Discussion

In this single-center study, patients with PIs exhibited significantly higher levels of C-reactive protein (CRP) and procalcitonin (PCT) than those without PIs. Before tNGS testing, the antibiotic utilization rate among patients with non-PIs was lower than that among patients with PIs, which was consistent with our previous findings (Huang et al., 2024). This finding can be attributed to the preliminary differentiation between infection and non-infection using traditional detection markers such as CRP and PCT. However, the inherent limitations of routine examinations prevent them from fulfilling the diagnostic requirements of clinical practice. With rapid advancements in laboratory testing technologies, tNGS, an emerging diagnostic tool, has gained robust pathogen identification capabilities and holds substantial promise for clinical applications.

This study demonstrated that, using the comprehensive judgment criteria as a reference, the pathogen detection sensitivity of BALF tNGS reached 88.8 % (95 % CI, 79.2%–94.4%), which was significantly higher than those of culture (22.5% [95% CI, 14.2–33.5%]) and CMTs (68.8% [95% CI, 58.5–76.6 %]). These findings were consistent with those of Guo et al. (2025), Liu et al. (2025). If the two methods are combined, the sensitivity can be further enhanced to 95.0 %. Additionally, the sensitivity of BALF tNGS was generally comparable to those reported by Chen et al. (2025) (87.33 %) and Li D. et al. (2025) (89.74 %), but lower than those observed by Guo et al. (2025) (95.83 %) and Gu et al. (2025) (97.73 %) and higher than that obtained with sputum tNGS (80.26 %) (Da et al., 2025). The variations in sensitivity observed across different studies can be attributed to methodological heterogeneity, including differences in sample size, specimen selection, and population characteristics. Furthermore, owing to the prolonged survival time of pathogen DNA in plasma, even when empirical anti-infective treatment is effective, antibiotics have a relatively minimal impact on the results of mNGS testing (Xie et al., 2020). In this study, although 86.3% of the patients with PIs in this cohort had received various antibiotics before testing, tNGS still exhibited high sensitivity, which was consistent with the results reported by Xu et al. (2024). These findings suggest that antibiotic use does not significantly affect the sensitivity of tNGS detection. However, despite demonstrating satisfactory sensitivity, tNGS exhibited a specificity of only 36.7 % (95% CI, 20.5%–56.1%), which was substantially lower than that of culture (96.7% [95% CI, 80.9–99.8%]). In previous studies, the specificity of tNGS for PIs ranged from 75.41% to 100% (Liu et al., 2025; Gu et al., 2025; Guo et al., 2025), substantially exceeding the value observed in this study. The primary reason for this discrepancy is likely the limited inclusion of non-infection-related cases in other comparable studies, which may have introduced greater bias into the results. Notably, the DNA viruses (particularly various human herpesviruses) and PJ identified in this study were predominantly clinically confirmed as non-pathogenic, thus obviating the need for any intervention. This finding is consistent with that reported by Kuang et al. (2024). Consequently, the relatively low specificity observed in this study may also be attributed to the high detection rate of non-pathogenic DNA/RNA viruses in patients without PIs (26.7%, 8/30). One notable pathogen was the human herpesvirus, as only one cytomegalovirus-positive case was considered clinically relevant for all 45 positive results. Therefore, clinicians must exercise caution when interpreting the test results. In particular, opportunistic pathogens such as herpesviruses, which are common latent infections with a tendency to reactivate, are typically pathogenic only in immunosuppressed patients (Grinde, 2013). Physicians should comprehensively integrate clinical data to avoid equating test reports directly with diagnostic conclusions, thereby preventing overdiagnosis and overtreatment. Despite this limitation, the diagnostic accuracy of tNGS was significantly higher than those of culture and CMTs. Moreover, the ROC curve analysis showed that tNGS had better diagnostic efficiency than CMTs in diagnosing PIs. However, owing to its unsatisfactory specificity, the AUC of tNGS (0.627) was relatively low, suggesting a modest diagnostic performance. Further optimization of the reporting criteria in the future is expected to reduce the false-positive rate and enhance the diagnostic efficiency of tNGS.

Our dataset indicates that community-acquired infections constitute the majority of PIs. tNGS demonstrated a significantly higher detection sensitivity than CMTs for single pathogens, mixed infections, bacteria, atypical pathogens, and viruses, particularly for the identification of atypical pathogens and RNA viruses. The use of traditional culture methods for *Mycoplasma* and *Chlamydia* is challenging because of the fastidious growth requirements of these microorganisms and because serum antibody detection exhibits suboptimal sensitivity. For RNA viruses such as influenza virus and SARS-CoV-2, throat swab nucleic acid or antigen testing serves as the conventional and cost-effective first-line method for detection. Clinicians frequently select appropriate laboratory tests based on specific clinical requirements. This selective approach may result in omitted tests and incomplete pathogen identification, particularly in mixed infections. As demonstrated in this study, in addition to mixed bacterial infections, the most prevalent type of mixed infection involves coinfections with viruses and bacteria. Compared with CMTs, tNGS can simultaneously detect multiple pathogens, including DNA and RNA microorganisms, providing comprehensive coverage of common clinical pathogens in a single test. tNGS has demonstrated significant advantages in the early diagnosis of viral infections, including high accuracy, rapid detection, effective differentiation of various viral types, and the ability to identify mixed infections. This can facilitate rapid identification of the target pathogen and enable precise targeted anti-infective treatments.

Notably, tNGS failed to exhibit any significant advantages for the detection of fungi. Further analysis indicated that in the diagnosis of fungal infections, CMTs predominantly depended on the detection of fungal antigens in the serum or BALF, specifically through the G, GM, and CrAg tests. However, as a positive G-test result cannot directly identify specific fungal species, its utility in guiding the selection of antifungal agents is limited. tNGS also showed no clear advantages in cryptococcal detection. For instance, in two cases of pulmonary cryptococcosis, both serum CrAg tests showed positive results, whereas only one tNGS result was positive, with one false-positive result. Therefore, CrAg testing is recommended as the primary diagnostic tool for patients with suspected cryptococcal infections (Tan et al., 2025). If the CrAg result is negative, as observed in a previous mNGS study (Huang et al., 2024), combining it with tNGS may enhance sensitivity. In addition, the sensitivities of tNGS and CMTs for the detection of MTB were comparable, in contrast to our previous findings on mNGS (Huang J. et al., 2023). This discrepancy can be attributed to the recent introduction of MTB DNA testing and GeneXpert MTB/RIF resistance testing assays in our laboratory, which substantially enhanced the diagnostic capability for tuberculosis. Owing to the limited number of pulmonary tuberculosis cases included in this retrospective study (6.4 %, 7/110), the data regarding the application of tNGS in detecting MTB may not be fully representative. Furthermore, our laboratory is currently only equipped to perform the GeneXpert MTB/RIF resistance testing assay, which does not fulfill the clinical demand for assessing resistance to other anti-tuberculosis medications (Liang and Tang, 2023). As the necessary conditions for performing growth-based phenotypic drug susceptibility testing (pDST) using mycobacterial cultures have not yet been established and given the absence of pDST as a reference standard, the pathogen drug resistance detection data obtained through tNGS were not analyzed. Therefore, the limited data obtained in this study are insufficient to provide robust evidence supporting the use of tNGS as the primary method for detecting MTB. Nevertheless, the small sample size demonstrated a high positivity rate (100 %) for MTB detection using tNGS, which was not inferior to that of CMTs. Therefore, tNGS may serve as a supplementary diagnostic tool in conjunction with traditional MTB testing. Furthermore, ordinary laboratories have limitations in identifying non-tuberculous mycobacteria (NTM). For instance, for acid-fast stain-positive specimens, these laboratories are incapable of differentiating MTB from NTM or determining the NTM subtype, which is crucial for guiding the development of appropriate medication plans. In contrast, tNGS demonstrates a strong capability for addressing this issue (Society of Clinical Microbiology of China International Exchange and Promotion Association for Medical and Healthcare, 2024).

As tNGS has significantly enhanced the sensitivity of pathogen detection and diagnostic efficiency, compared with traditional culture methods (13 vs. 48 h) and CMTs, it provides crucial guidance for the development of clinical strategies. In this study, tNGS results were used to adjust the clinical treatment plans for 45.5 % (50/110) of the patients. The adjustments primarily involved initiating targeted anti-infective therapy after identifying the causative pathogen (16.4 %, 18/110) and escalating antibiotic regimens (19.1%, 21/110). A previous study demonstrated that the rate of antibiotic escalation in patients who underwent detection using mNGS was lower than that in patients who underwent detection using CMTs (19.0 % vs. 26.8 %), indicating that mNGS testing is associated with a decreased likelihood of antibiotic escalation (Yan et al., 2024). In this study, the rate of antibiotic escalation was relatively low, which may be attributed to the positive effects of the tNGS on clinical decision-making. Empirical anti-microbial treatments have been widely adopted in clinical practice. However, without robust support from subsequent laboratory test results, the variability and complexity of the condition may lead to frequent changes in therapeutic agents and an inappropriate escalation of antibiotic use during the diagnostic and treatment processes. Based on the advantages of tNGS, when empirical antibacterial therapy already encompasses the pathogens detected by tNGS, the attending physician is more likely to decide against escalating the antibiotic regimen based on the clinical context. Therefore, although 25.5 % of the patients in this study did not undergo adjustment of their anti-infection regimens following tNGS, this does not imply that tNGS provided no therapeutic guidance to these patients. In contrast, the timely and comprehensive microbial results delivered by tNGS enable clinical teams to determine the final treatment plan more rapidly and precisely, minimizing unnecessary adjustments and escalations in antibiotic use (Liang et al., 2022). A typical example is infections by *Chlamydia*, a pathogen that is not easily detectable in conventional laboratories. In this study, five of the six patients infected with *Chlamydia psittaci* confirmed by tNGS were accurately treated empirically before tNGS testing. However, in two cases, although the symptoms improved after empirical treatment with moxifloxacin and levofloxacin, the lesions in both lungs were not notably absorbed. Based on the tNGS results, the original treatment plan was maintained and the patients were eventually cured, avoiding unnecessary escalation of antibiotics. Notably, the other three cases exhibited persistent fever. Based on the tNGS results, all the three patients were successfully treated by supplementing doxycycline with previously administered quinolone drugs (levofloxacin or moxifloxacin). Consequently, the patient’s body temperature normalized. Although these three cases were categorized as involving antibiotic escalation because combination therapy was used, this approach averted the need for higher-level antibiotics, such as enzyme inhibitor combinations or carbapenems, thereby preventing potential deviations in the treatment direction. In addition, only one case in this study involved the de-escalation of antibiotic therapy (excluding eight patients who were confirmed to have no infection and thus discontinued antimicrobial agents), which represents a significantly low frequency. This finding is consistent with the results of the mNGS study reported by Liang et al. (2022) and may be primarily attributed to the challenges associated with interpreting the results, such as those encountered in mNGS. Although tNGS can detect a wide range of opportunistic pathogenic and normal respiratory tract-colonizing bacteria while screening for target pathogens, it currently shares a limitation with mNGS: its inability to effectively differentiate between pathogenic and colonizing microorganisms. This challenge substantially interferes with the decision-making process regarding the de-escalation of antibiotic therapy. Inconsistencies were also observed between tNGS and CMTs results. In this study, approximately 30 % of the treatment regimens were determined based on CMTs. Specifically, clinicians tend to adopt relatively conservative antibiotic regimens guided by CMT results rather than relying solely on tNGS results to downgrade the treatment. This further indicates that tNGS can serve as a valuable complement to CMTs, rather than completely substituting conventional laboratory techniques.

Nine patients died, and the remaining patients showed stable condition, improvement, or complete recovery. Five patients received escalated antibiotic therapy based on the results of tNGS testing. Among these patients, three initially experienced controlled infections and clinical improvement; however, their infections subsequently recurred and deteriorated, ultimately resulting in treatment failure. Analysis of the underlying causes showed that all five patients were critically ill, had multiple comorbidities, and were infected with multidrug resistant bacteria. These factors collectively contribute to the limited selection of effective therapeutic options and substantially increase treatment complexity. Despite these limitations, the rapid detection and high sensitivity of tNGS allow the identification of pathogens at an earlier stage and show that it holds significant promise for guiding anti-infection treatments for severe pneumonia (Zhang et al., 2024).

However, this study has certain limitations that warrant acknowledgment. First, this was a single-center, retrospective study with a relatively small sample size. This may have resulted in data bias. However, the sample size of this study was significantly larger than those of similar studies [*n* = 8 (Guo et al., 2025); *n* = 50 (Liu et al., 2024); and *n* = 61 (Zhang et al., 2023)] or comparable with those of other relatively larger studies [*n* = 130 (Zhang et al., 2024)] and [*n* = 150 (Chen et al., 2025)]. Notably, some findings were inconsistent with those of the previous studies, particularly in terms of specificity, which was relatively low. Therefore, additional validation through studies with larger sample sizes or multicenter collaborations is required. Second, as our institution is not a specialized tuberculosis hospital, it lacks the capacity to perform MTB and NTM cultures. This limitation contributed to the low positive detection sensitivity of traditional culture methods. Third, given that some patients commenced targeted anti-infection therapy immediately following rapid identification of the target pathogen through tNGS, certain conventional testing procedures, such as nucleic acid tests for influenza virus, SARS-CoV-2, and mycoplasma in the BALF, are no longer performed. This may have reduced the actual detection sensitivity of CMTs. Fourth, tNGS frequently identifies a substantial number of non-pathogenic microorganisms. This technology lacks the capability to differentiate between colonizing and pathogenic bacteria, necessitating a high level of comprehensive analytical skills among clinicians for an accurate distinction. Fifth, although tNGS can simultaneously detect hundreds of pathogens, its reliance on predefined panels limits its ability to identify newly emerging or exceedingly rare pathogens. mNGS offers distinct advantages in such scenarios. Sixth, although its detection cost has significantly reduced, this emerging technology with broad pathogen coverage remains higher than that of most CMTs. Furthermore, most medical institutions depend on third-party platforms for testing, and the establishment of in-house platforms continues to present numerous challenges. Finally, a previous study demonstrated that tNGS possesses a robust capability for detecting antibiotic resistance and exhibits high accuracy in predicting antibiotic resistance (Li Y. et al., 2025). However, due to the limited number of positive cases identified through traditional culture in this study, statistical analysis of the tNGS data for the detection of antibiotic resistance genes was not performed. Therefore, the sample size should be increased in future studies to further evaluate the performance of tNGS.

In summary, the sensitivity and accuracy of tNGS were better than those of culture and CMTs. The tNGS results are critically associated with the development of clinical treatment plans for most patients. Generally, tNGS can be used as a rapid and accurate auxiliary diagnostic method for PIs in the following scenarios: when CMTs yield negative results in non-severe patients; when clinical suspicion of infection is high but etiological evidence is difficult to obtain through CMTs; when differentiation between MTB and NTM is required; and when there is existing pathogenic evidence, but the therapeutic response is suboptimal, raising the suspicion of mixed or secondary infection (Society of Clinical Microbiology of China International Exchange and Promotion Association for Medical and Healthcare, 2024). As tNGS has certain limitations, such as the potential for false-positive results and limited ability to differentiate pathogenicity among detected organisms, CMTs are indispensable. In the future, combining tNGS with artificial intelligence-based interpretation or machine learning may improve specificity and colonization-pathogen differentiation.

## Data Availability

The data presented in the study are deposited in the National Center for Biotechnology Information repository, accession number PRJNA1336118.
