# Supplementary material for: Targeted next-generation sequencing using bronchoalveolar lavage fluid samples for diagnosing pulmonary infections: a single-center retrospective study
Source: Front Microbiol. 2025 Oct 13;16:1671819. doi: 10.3389/fmicb.2025.1671819 (PMC12554695; doi:10.3389/fmicb.2025.1671819)
Supplement: Supplementary file 1 [file Data_Sheet_1.docx]

Supplementary Table 1. Pathogens detected by tNGS in patients with pulmonary infections

| Pathogen species (n = 71) | n (%) |
| --- | --- |
| Single pathogen  Single bacterium  General bacterium^a^  *Mycobac**terium tuberculosis* complex  Non-tuberculous *mycobacteria*  *Mycobacterium* *abscessus*  Atypical pathogen  *Chlamydia psittaci*  *Chlamydia pneumoniae*  *Mycoplasma pneumoniae*  Single fungi  *Pneumocystis jirovecii*  *Cryptococcus neoformans*  *Talaromyces marneffei*  *Aspergillus fumigatus*  Single virus  SARS-Cov-2  Influenza A virus  Human metapneumovirus  Mixed pathogens ^b^ | 50 (70.4)  40 (80.0)  25 (62.5)  6 (15.0)  1 (2.5)  1 (100)  8 (20.0)  5 (62.5)  1 (12.5)  2 (25.0)  6 (12.0)  3 (50.0)  1 (16.7)  1 (16.7)  1 (16.7)  4 (8.0)  1 (25.0)  2 (50.0)  1 (25.0)  21 (29.6) |

Abbreviations: SARS-Cov-2, severe acute respiratory syndrome coronavirus-2; tNGS, targeted metagenomic next-generation sequencing.

Data are presented as n (%) .

^a^ General bacteria included *Pseudomonas aeruginosa* (seven cases)*, Haemophilus influenzae* (five cases)*,* *Streptococcus pneumoniae* (three cases)*,* *Acinetobacter baumannii* (two cases)*,* *Enterobacter cloacae* complex (two cases), *Klebsiella pneumoniae* (one case)*, Moraxella catarrhalis* (one case)*, Acinetobacter nosocomialis* (one case), *Streptococcus mitis* (one case), *Tannerella forsythia* (one case), *Porphyromonas gingivalis* (one case).

^b^ Mixed pathogens (≥ two pathogens), including bacteria, fungi, and viruses.
