# Supplementary material for: Targeted next-generation sequencing using bronchoalveolar lavage fluid samples for diagnosing pulmonary infections: a single-center retrospective study
Source: Front Microbiol. 2025 Oct 13;16:1671819. doi: 10.3389/fmicb.2025.1671819 (PMC12554695; doi:10.3389/fmicb.2025.1671819)
Supplement: Supplementary file 2 [file Data_Sheet_2.docx]

Supplementary Table 2. Mixed pathogens detected by tNGS in patients with pulmonary infections

| Mixed pathogen (n = 21) | n (%) |
| --- | --- |
| Two types of pathogens  *Klebsiella pneumoniae* and *Pseudomonas aeruginosa*  *Acinetobacter baumannii* and *Serratia marcescens*  *Acinetobacter baumannii* and *Klebsiella pneumoniae*  *Streptococcus pneumoniae* and *Haemophilus influenzae*  *Streptococcus pneumoniae* and *Acinetobacter nosocomis*  *Streptococcus pneumoniae* and *Aspergillus niger*  *Streptococcus pneumoniae* and Influenza A virus  *Streptococcus pneumoniae* and Influenza B virus  *Streptococcus mitis* and Human metapneumovirus  *Klebsiella pneumoniae* and Influenza A virus  *Klebsiella pneumoniae* and SARS-Cov-2  *Pneumocystis jirovecii* and SARS-Cov-2  *Haemophilus influenzae* and SARS-Cov-2  Influenza A virus H3N2 subtype and SARS-Cov-2  *Staphylococcus aureus* and Influenza A virus H3N2 subtype  *Mycobac**terium tuberculosis* complex and Influenza A virus  *Chlamydia psittaci* and Influenza A virus  *Moraxella catarrhalis* and Cytomegalovirus  Three types of pathogens  *Streptococcus pneumoniae*, *Aspergillus fumigatus*, and Influenza A virus  *Klebsiella pneumoniae*, *Streptococcus pneumoniae*, and *Aspergillus nidulans* | 19 (90.5)  2  1  1  1  1  1  1  1  1  1  1  1  1  1  1  1  1  1  2 (9.5)  1  1 |

Abbreviations: SARS-Cov-2, severe acute respiratory syndrome coronavirus-2;

tNGS, targeted metagenomic next-generation sequencing.

Data are presented as n (%) .
