# Supplementary material for: Targeted next-generation sequencing using bronchoalveolar lavage fluid samples for diagnosing pulmonary infections: a single-center retrospective study
Source: Front Microbiol. 2025 Oct 13;16:1671819. doi: 10.3389/fmicb.2025.1671819 (PMC12554695; doi:10.3389/fmicb.2025.1671819)
Supplement: Supplementary file 3 [file Data_Sheet_3.docx]

Supplementary Table 3: Changes to therapy based on tNGS results in five deceased patients

| Patient No. | tNGS testing | Empirical treatment prior to tNGS testing | Modifications to treatment plans following tNGS testing | Prognosis |
| --- | --- | --- | --- | --- |
| 1  2  3  4  5 | *Acinetobacter baumannii*  *Klebsiella pneumoniae*,, *Streptococcus pneumoniae*, and *Aspergillus nidulans*  *Acinetobacter baumannii* and *Serratia marcescens*  *Acinetobacter baumannii* and *Klebsiella pneumoniae*  *Klebsiella pneumoniae* and Influenza A virus | Imipenem-Cilastatin and Linezolid  Moxifloxacin and Cefperazone-Sulbactam  Meropenem and Polymyxin B  Ceftazidime-Avibactam and Aztreonam  Levofloxacin and Cefperazone-Sulbactam | Tigecycline and Polymyxin B  Tigecycline, Polymyxin B, and Amphotericin B liposome  Tigecycline and Polymyxin B  Tigecycline and Polymyxin B  Meropenem and Oseltamivirr | Death  Death due to a secondary infection  Death  Death due to a secondary infection  Death due to repeated aspiration |

Abbreviations: tNGS, targeted metagenomic next-generation sequencing.
