# Supplementary figures and images for: Targeted next-generation sequencing using bronchoalveolar lavage fluid samples for diagnosing pulmonary infections: a single-center retrospective study
Source: Front Microbiol. 2025 Oct 13;16:1671819. doi: 10.3389/fmicb.2025.1671819 (PMC12554695; doi:10.3389/fmicb.2025.1671819)

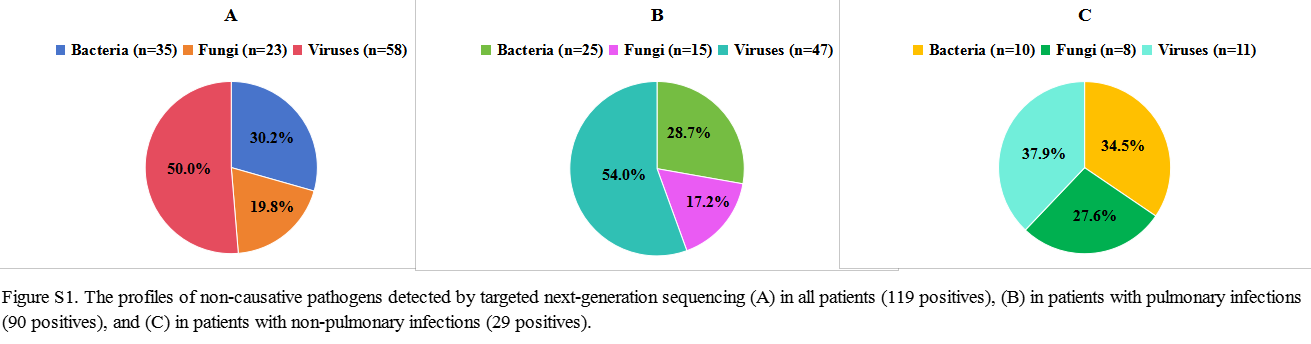

Supplement: Supplementary file 5 [file Image_1.tif]

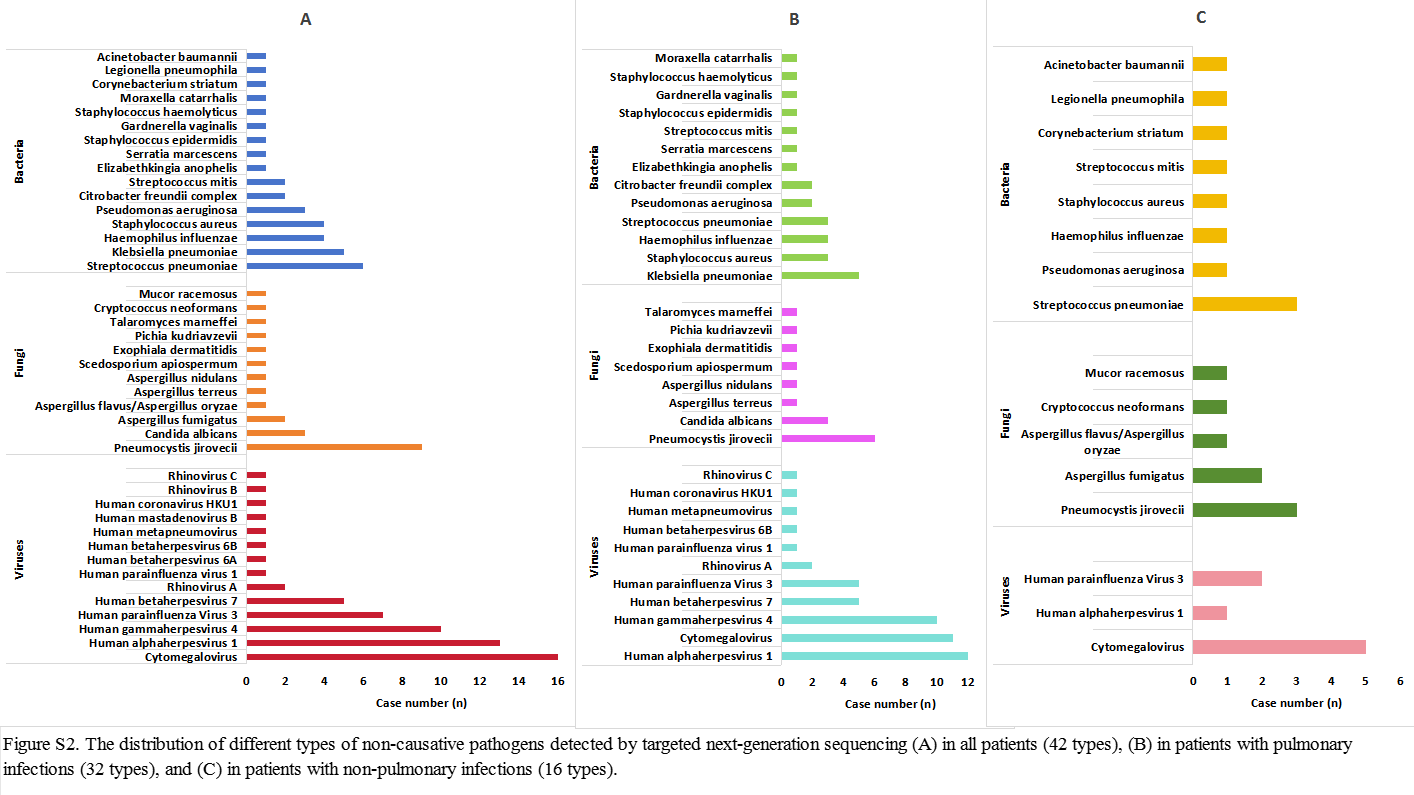

Supplement: Supplementary file 6 [file Image_2.tif]
